# Cerebral oxygenation monitoring using near infrared spectroscopy during one-lung ventilation in adults

**DOI:** 10.4103/0972-9941.45206

**Published:** 2008

**Authors:** Joseph D Tobias, Garry A Johnson, Saif Rehman, Robert Fisher, Norman Caron

**Affiliations:** 1 Department of Anaesthesiology University of Missouri, Columbia, Missouri; 2 Department of Paediatrics, University of Missouri, Columbia, Missouri; 3 Department of Surgery, University of Missouri, Columbia, Missouri

**Keywords:** Cerebral oxygenation, near infrared spectroscopy, one-lung ventilation, thoracic surgery, thoracoscopy

## Abstract

**BACKGROUND:**

Changes in oxygenation occur during one-lung ventilation (OLV) due to intrapulmonary shunt. Although arterial oxygenation is generally adequate, there are no studies evaluating the effect of these changes on cerebral oxygenation.

**MATERIALS AND METHODS:**

Cerebral oxygenation (rSO_2_), heart rate (HR), blood pressure (BP), oxygen saturation (SaO_2_), and end-tidal carbon dioxide (ETCO_2_) were prospectively monitored during OLV in adults. Cerebral oxygenation was monitored using near infrared spectroscopy. No clinical decisions were made based on the rSO2 value. BP and HR were the inspired oxygen concentration was adjusted as needed to maintain the SaO_2_ ≥ 95%.

**RESULTS:**

The study cohort included 40 adult patients. 18,562 rSO_2_ values were collected during OLV. The rSO_2_ was ≥ baseline at 3,593 of the 18,562 data points (19%). The rSO2 was 0-9 ≤ baseline in 7,053 (38%) of the readings, 10-19 ≤ baseline in 4,084 (22%) of the readings, and 20-29 ≤ baseline in 3,898 (21%) of the readings. 2,599 (14%) of the rSO_2_ values were less than 75% of the baseline value. Thirteen patients (32.5%) had at least one rSO2 value that was less than 75% of the baseline. Eight patients (20%) had rSO_2_ values less than 75% of baseline for ≥ 25% of the duration of OLV. These patients were older (63.7 ± 10.2 vs 54.6 ± 9.8 years, *P*<0.025), weighed more (95.8 ± 17.4 vs 82.6 ± 14.6 kgs, *P*=0.038), and were more likely to be ASA III vs II (7 of 8 versus 25 of 32, relative risk 1.75) than the remainder of the cohort.

**CONCLUSIONS:**

Significant changes in rSO2 occur during OLV for thoracic surgical procedures. Future studies are needed to determine the impact of such changes on the postoperative course of these patients.

## INTRODUCTION

One-lung ventilation (OLV) is frequently used during open and endoscopic thoracic procedures to eliminate lung movement and improve surgical visualisation. With newer thoracoscopic procedures, effective OLV has become mandatory to facilitate completion of the procedure and avoid the need for open thoracotomy. During OLV, even in the presence of effective hypoxic pulmonary vasoconstriction, intrapulmonary shunting still occurs, resulting in alterations in systemic oxygenation. These modest decreases in systemic oxygenation are accepted with the assumption that tissue oxygenation remains adequate. However, there are limited data examining the end-organ effects of the alterations in respiratory and cardiovascular function which may occur during OLV.

Near infrared spectroscopy (NIRS), otherwise known as cerebral oximetry, is a non-invasive device that uses infrared light to estimate brain tissue oxygenation (rSO_2_).[1] The potential use of NIRS as a means of monitoring cerebral oxygenation was first suggested by Jobsis in 1977.[2] In a technique similar to pulse oximetry, NIRS uses infrared light to penetrate living tissue and estimate brain tissue oxygenation by measuring the absorption of infrared light by tissue chromophores such as haemoglobin. After the infrared light penetrates living tissue, the relative absorption of the different wavelengths is dependent on the concentration of the various hemoglobin species (unoxygenated *vs* oxygenated). Based on the relative absorption of the infrared light at various wavelengths, the specific concentration of the haemoglobin species can be determined using a modification of the Beer-Lambert law.[1–3] Previous studies have suggested that decreases in cerebral oxygenation as measured by NIRS may occur even without changes in routine intraoperative monitoring techniques including heart rate (HR), blood pressure (BP), and oxygen saturation measured by pulse oximetry (SaO_2_).[4–7] Additionally, it has been demonstrated that these episodes of cerebral oxygen desaturation may correlate with postoperative neurocognitive dysfunction and that monitoring and treating these episodes may decrease the incidence of postoperative neurocognitive dysfunction.[6,7] To date, there are no studies evaluating changes in rSO_2_ which may occur during OLV. The current study prospectively monitored cerebral oxygenation using NIRS during OLV in adults.

## MATERIALS AND METHODS

The study was approved by the University's Institutional Review Board and informed consent was obtained from the patients. Patients 18 years of age and greater scheduled for thoracic surgery requiring OLV were considered eligible for inclusion. Patients with a pre-existing oxygen requirement or those requiring preoperative mechanical ventilation were not considered eligible. The choice of anaesthetic agents was not standardised and was at the discretion of the anaesthesia team. Cerebral oxygenation (rSO_2_) was continuously monitored using near infrared spectroscopy (INVOS 3100A, Somanetics Corporation, Troy, MI) with a single sensor placed on the right side of the patient's forehead with its caudad border approximately 1 cm above the patient's eyebrow and the medial edge at the midline according to the manufacturer's guidelines. The rSO_2_ value was recorded every 10 sec during the procedure. The anaesthesia team was not allowed to view the rSO_2_ value during the procedure. Oxygen saturation measured by pulse oximetry (SaO_2_), end-tidal carbon dioxide (ETCO_2_), BP, and HR were continuously monitored and the values recorded every 5 min. OLV was provided using either a double lumen endotracheal tube or a bronchial blocker. The surgical procedures were performed in either the right or left lateral decubitus position. During the procedure, the BP was maintained within 20% of baseline by the administration of fluids or the direct acting α-adrenergic agonist, phenylephrine, for a low BP and by increasing the concentration of the inhalational anaesthetic agent or the administration of additional opioid (fentanyl) for a high BP. The inspired oxygen concentration (F_i_O_2_) was adjusted as needed with a goal of maintaining the oxygen saturation at ≥ 95%. During OLV, the tidal volume was decreased to 6-8 mL/kg and the ventilation rate adjusted as needed to maintain the PaCO_2_ at 35-40 mmHg.

The rSO_2_ values obtained prior to the start of OLV were averaged for each patient and then used as the patient's baseline for subsequent analysis. The values obtained during OLV were then compared to the baseline values and categorised as an increased in rSO_2_ from baseline or as an absolute decrease from baseline of 0-9, 10-19, 20-29 or ≥ 30. The number of values representing an rSO_2_ decrease to less than 75% and 80% of the baseline was also determined. The demographics (age, weight, gender, ASA designation), hemodynamic values (HR, BP) and SaO_2_ values of patients with an rSO_2_ value less than 75% of baseline were compared to those of patients without cerebral desaturation using a non-paired t-test and Fisher's exact test with a contingency Table. The data are presented as the mean ± SD.

## RESULTS

The study cohort included 40 adults ranging in age from 38 to 78 years of age and in weight from 59 to 122 kg. Eight were ASA II and 32 were ASA III. Open thoracotomy was performed in 28 and thoracoscopy in 12. The baseline rSO_2_ varied from 51 to 78 (65 ± 11). The duration of OLV varied from 45 to 98 min (78.8 ± 20.6 minutes). A total of 18,562 rSO_2_ values were collected during OLV. The rSO_2_ was ≥ baseline at 3,593 of the 18,562 data points (19%). For the remaining 14,969 data points, the rSO_2_ value was ≤ baseline [Table 1]. The rSO_2_ was 0-9 ≤ baseline in 7,053 (38%) of the readings, 10-19 ≤ baseline in 4,084 (22%) of the readings, and 20-29 ≤ baseline in 3,898 (21%) of the readings. No rSO_2_ values was 30 ≤ the average baseline values. A total of 4,459 of the rSO_2_ values (24%) were less than 80% of the baseline value and 2599 (14%) were less than 75% of the baseline value. Twenty-one patients (52.5%) had at least one rSO_2_ value that was less than 80% of the baseline and 13 patients (32.5%) had at least one rSO_2_ value that was less than 75% of the baseline. Eight patients (20%) had rSO_2_ values less than 75% of baseline for more than 25% of the duration of OLV.

**Table 1:** Distribution of cerebral oxygenation values during one-lung ventilation

Absolute change of rSO2 from baseline	Number of values	Percentage of total
More than baseline	3,527	19
0-9 less baseline	7,053	38
10-19 less than baseline	4,084	22
20-29 less than baseline	3,898	21
> 30 less than baseline	0	0

Patients with prolonged decreases in rSO_2_ to less than 75% of the baseline value for 25% or more of the duration of OLV (n=8) were older (63.7 ± 10.2 *vs* 54.6 ± 9.8 years, p<0.025), weighed more (95.8 ± 17.4 versus 82.6 ± 14.6 kg, p=0.038), and were more likely to be ASA III *vs* II (7 of 8 *vs* 25 of 32, relative risk 1.75) than the remainder of the cohort (n=32). There was no difference between the two groups in regards to gender, duration of OLV, and the intraoperative SaO_2_, HR, BP, or ETCO_2_ values.

## DISCUSSION

The potential for postoperative neurocognitive dysfunction and its impact on the postoperative course has gained recent attention over the past few years.[8] Although likely multi-factorial, recent data suggest that changes in cerebral oxygenation as assessed by NIRS may be a risk factor.[6,7] In a prospective trial, 122 elderly patients undergoing major abdominal surgery under general anaesthesia were randomly assigned to a treatment group in which the rSO_2_ value was monitored and maintained at ≥ 75% of the preinduction values or a monitor only group in which cerebral oximetry was monitored, but not visible to the anaesthesiologist.[6] Cerebral oxygen desaturation (≤ 75% of baseline) was observed in 11 patients of the treatment group (20%) and 15 patients in the control group (23%). The treatment protocol to treat cerebral oxygen desaturation included ensuring adequate ventilation, checking head position, increasing the F_i_O_2_, increasing the PaCO_2_, and increasing the blood pressure with fluids or vasoconstricting medications. Control patients with cerebral oxygen desaturation had lower mini-mental status examination (MMSE) scores on postoperative day #7, longer PACU stays (median: 47 minutes *vs* 25 min, *P*=0.01) and longer hospital stays (median: 24 days *vs* 10 days, *P*=0.007) when compared with the treatment patients. More recently, Murkin *et al*. randomized 200 adults undergoing coronary artery bypass grafting to either a treatment group where the rSO_2_ was monitored and treated or a monitor only group.[7] There was an increased incidence of prolonged cerebral desaturation in the monitor only group (6 *vs* 0, *P*=0.014) as well as a longer stay in the Intensive Care Unit (*P*=0.029). Additionally, there was an increased incidence of major end-organ morbidity and mortality defined as death, the need for mechanical ventilation for > 48h, stroke, myocardial infarction, or need for re-exploration (*P*=0.048).

In our prospective evaluation, we noted as with previous studies that changes in rSO_2_ frequently occurred without changes in haemodynamic or ventilatory parameters. Therefore, monitoring with standard ASA monitors fails to identify these periods of cerebral desaturation. Thirteen of the 40 patients (32.5%) had at least one rSO_2_ value that was less than 75% of the baseline, which using the criteria of Casati *et al*, may place these patients at higher risk for postoperative neurocognitive dysfunction. Additionally, eight patients or 20% of the current cohort had rSO_2_ values that were ≤ 75% of baseline for more than 25% of the duration of OLV or approximately 20 min. We noted no correlation of these changes with HR, BP, ETCO_2_, or SaO_2_ values, findings similar to those reported by other investigators. The incidence of significant cerebral desaturation was higher in our current cohort of patients than demonstrated in previous studies as almost one-third of our cohort had at least one rSO_2_ value that was ≤ 75% of baseline compared to 20% in the study of Casati *et al* and 9% in our previous study in adults during laparoscopic procedures.[4,6] One explanation for the increased incidence of cerebral desaturation in the current cohort may be that rSO_2_ values were recorded every 10 sec compared to other studies where values were recorded at intervals varying from 5 to 30 minutes. In the current cohort, each rSO_2_ value that was obtained during OLV (n=18,562) was compared to the baseline rather than averaging values over a period of time. Alternatively, the use OLV may place these patients at higher risk for cerebral desaturation due to the presence of intrapulmonary shunt and lower PaO_2_ values during the procedure. As with our previous study in adult laparoscopic surgery,[4] we did note that patients who experienced rSO_2_ values ≤ 75% of baseline were older, weighed more, and were more likely to be ASA category III.

There are specific limitations of the current study. We did not separate the patients into treatment and non-treatment groups to determine if treating the rSO_2_ decreases would alter postoperative outcome. Our intent was only to perform an observational study to determine if cerebral desaturation occurs during OLV as with other major surgical procedures. Additionally, we did not investigate the effect of intraoperative episodes of cerebral desaturation on postoperative outcome and since the anaesthetic technique was not strictly controlled, we can make no comment regarding the anaesthetic technique and its impact on cerebral desaturation. Based on the relatively high incidence of decreases in rSO_2_ during thoracic procedures using OLV, we would suggest that future studies are needed to address these issues.
